# qRT-PCR evaluation of the transcriptional response of zebra mussel to heavy metals

**DOI:** 10.1186/s12864-015-1567-4

**Published:** 2015-05-06

**Authors:** Joaquim Jaumot, Anna Navarro, Melissa Faria, Carlos Barata, Romà Tauler, Benjamín Piña

**Affiliations:** Department of Environmental Chemistry, IDAEA-CSIC, Jordi Girona 18-26, Barcelona, 08034 Spain

**Keywords:** qRT-PCR, Chemometric analysis, *Dreissena polymorpha*, Metal exposure, Gene networks, Oxidative stress pathways, Transcriptional regulation

## Abstract

**Background:**

The transcriptional response of adult zebra mussels (*Dreissena polymorpha*) to heavy metals (mercury, copper, and cadmium) was analyzed by quantitative Real-Time Polymerase Chain Reaction (qRT-PCR) to study the coordinated regulation of different metal-, oxidative stress- and xenobiotic defence-related genes in gills and digestive gland. Regulatory network analyses allowed the comparison of this response between different species and taxa.

**Results:**

Chemometric analyses allowed identifying the effects of these metals clearly separating control and treated samples of both tissues. Interactions between the different genes, either in the same or between both tissues, were analysed to identify correlations and to propose stress-related genes’ regulatory networks. These networks were finally compared with existing data from human, mouse, zebrafish, Drosophila and the roundworm to evaluate their mechanistically-known response to metals (and to stressors in general) with the correlations observed in the still poorly-known, invasive zebra mussel.

**Conclusions:**

Our analyses found a general conservation of regulation genes and of their interactions among the different considered species, and may serve as a guide to extrapolate regulatory data from model species to lesser-known environmentally (or medically) relevant species.

**Electronic supplementary material:**

The online version of this article (doi:10.1186/s12864-015-1567-4) contains supplementary material, which is available to authorized users.

## Background

The survival of organisms to the ever-changing environmental conditions depends on their capacity to cope with the multiple stressors they are exposed to. The coordinated activation of different stress mechanisms is a fundamental element of the overall response to pollutants and to other potentially deleterious external inputs [1]. On the very roots of these coordinated responses lies an intricate network of regulatory elements at genetic level, adapting the cell metabolism first to survive to the sudden external changes and afterwards to acclimate to the new, and often unfavourable, conditions. DNA microarrays (and ultimately, high-throughput sequencing) are the standard instrumental technique to monitor changes in gene expression of essentially all genes [2]. There has been an increasing interest in the literature on chemometric data pre-treatment and data analysis methods dealing with microarray data [3,4]. However, other instrumental techniques can also monitor gene expression variations in multiple samples. One of these techniques is the quantitative Real-Time Polymerase Chain Reaction (qRT-PCR) that allows detecting and quantifying target DNA molecules [5]. The main advantage of this method is that it allows quantitation of changes in mRNA levels (usually related to gene expression variations) in a very wide range of values (>10^7^-folds), resulting in assays with very high sensibility, selectivity, and reproducibility [5,6]. In addition, high-throughput systems allow analysing hundreds of transcripts for many samples simultaneously, which allow obtaining a large quantity of data in a single experiment. Different studies using qRT-PCR have appeared in the recent years in the literature, studying the response of different organisms at the gene expression level in so diverse research fields such as drug discovery, cancer research, environmental assays [7-11]. However, the analysis of qRT-PCR data by means of chemometric methods has not yet received the same attention as the analysis of DNA microarrays data, and only a small number of studies about this topic can be found in the literature [12-14].

In this work, variations in the gene expression of the zebra mussel (*Dreissena polymorpha*) associated with environmental stresses, such as the presence of pollutants, are investigated by means of chemometric analysis of qRT-PCR data. This freshwater mussel species has been selected due to its invasive character, which brought it to expand from its natural geographic distribution in the Caspian and Black seas to a real worldwide distribution in the last few decades [15]. In some places, this expansion has led to large infestations with significant economic and environmental consequences [15]. One of these colonisations has occurred in the Ebro river basin (North East Spain) where it has become a danger to native species [16]. Zebra mussel is the only freshwater bivalve that can be legally collected for environmental monitoring. This circumstance, together with the known ability of the zebra mussel to bioaccumulate contaminants, has increased the interest of this species as a sentinel notably for biomonitoring purposes and quality control of water ecosystems [17-23].

As a training dataset, we used previously reported qRT-PCR data from gills and digestive glands of adult zebra mussels exposed to different heavy metals concentrations (copper, cadmium and mercury) [22]. Several multivariate data analysis approaches have been tested with the final goal of monitoring and distinguishing between effects caused by heavy metals and exposure time, and with the goal of identifying the genes most affected by the investigated pollutants. Finally, biological interpretation has been obtained from a comparison with genetic and regulatory networks in different model species.

## Results

**Figure 1:**
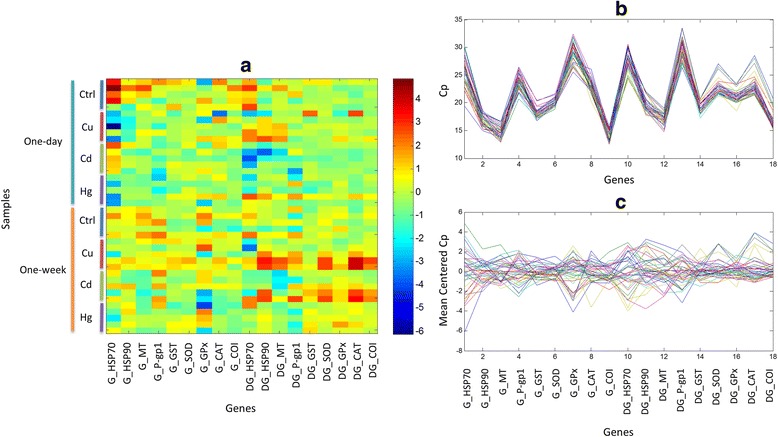
Representation of qRT-PCR experimental data. **a)** Heat map representation of the mean-centered data (40 samples and 18 genes according to Additional file 3 Figure S2). Plot of the experimental data **b)** before, and **c)** after mean-centering.

Figure 1 shows the mean-centred data matrix composed of 40 samples and 18 genes. The visual representation of this data matrix did not show any feature easy to be interpreted. For instance, the heat map representation of the data (Figure 1a) did not allow gathering any relevant information about possible relationships between genes and samples directly. Therefore, different multivariate data analysis methods were tested to investigate relationships between genes and samples.

### Graphical investigation of gene correlations

**Figure 2:**
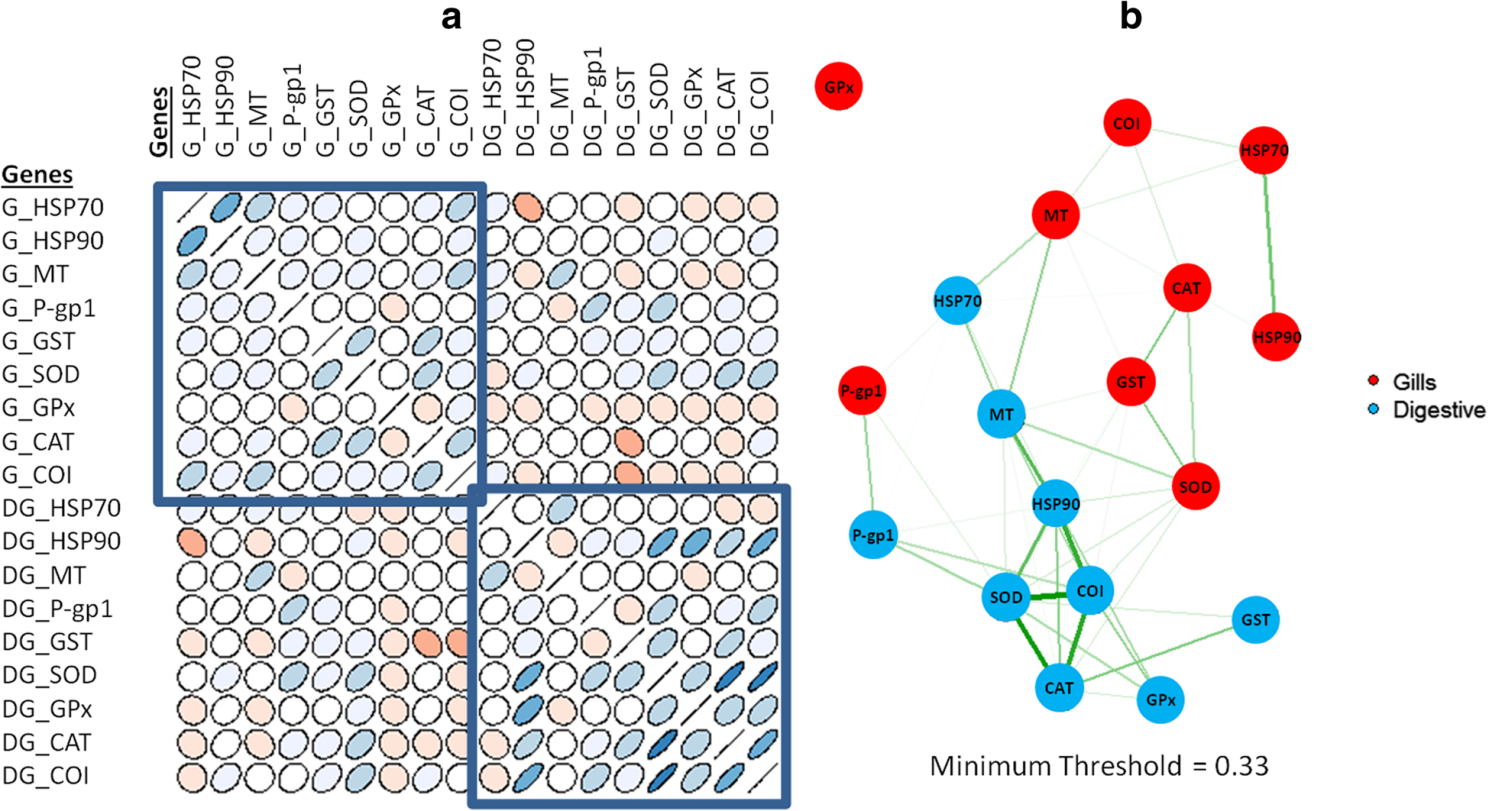
Correlation coefficient based analysis. **a)** Correlation map based on ellipse codification (legend: blue: positive correlation, red: negative correlation, eccentricity scaled to the correlation value), and **b)** Gene network corresponding based on the correlation matrix (width of the lines is proportional to the correlation coefficient value).

Relationships between genes from the same and different tissues were investigated. Correlation matrix plot between the 18 considered genes is shown in Figure 2a. From this representation, a preliminary interpretation can be obtained. First, it is worth to focus the attention on correlations between genes from the same tissue. Close to the diagonal of the correlation matrix (samples 1 to 9 for gills, and samples 10 to 18 for digestive gland), genes had positive correlation values whereas genes placed farther from the diagonal (corresponding to the other tissue) showed either no correlation or inverse (negative) correlation. Next, correlation values between SOD, CAT and COI genes of digestive glands showed high positive correlations, as well as for gill tissues, but of lower intensity.

Correlation diagrams showed relationships already commented above, but a deeper analysis of the data was attempted to extract more information. A representation of the correlation matrix as a gene network is shown in Figure 2b. This plot shows relationships between genes in a clearer and quicker way. Interpretation of this network diagram demonstrated that there were no relevant relations between gene expressions of the two considered tissues (correlations between genes from different tissues were weak). In contrast, relationships between genes from the same tissue were strong. So, in digestive glands a cluster of genes with strong correlations included SOD, COI, CAT and, also, HSP90. Other genes such as GPx, GST or MT showed weaker correlations. For gill tissue genes, correlation between SOD and CAT was also high, although, in this case, correlation with GST was greater than that for COI. It is also worth to mention the behaviour of the P-gp1 gene. In digestive gland tissue, this gene showed a weak correlation with the SOD-COI-CAT cluster, wherein of gills, this correlation was inappreciable. Conversely, there was a strong correlation between the expressions of these genes in both tissues, which was the only case of such an inter-tissue correlation observed for this dataset. Finally, the behaviour of the gill GPx gene did not show any correlation with any other of the considered genes from either tissue.

Similar results were obtained when experimental data were analysed by means of unsupervised hierarchical clustering. In this case, no previous information was provided to the algorithm and genes were clustered iteratively in an agglomerative manner using Ward’s [24] and Euclidian distance methods.

**Figure 3:**
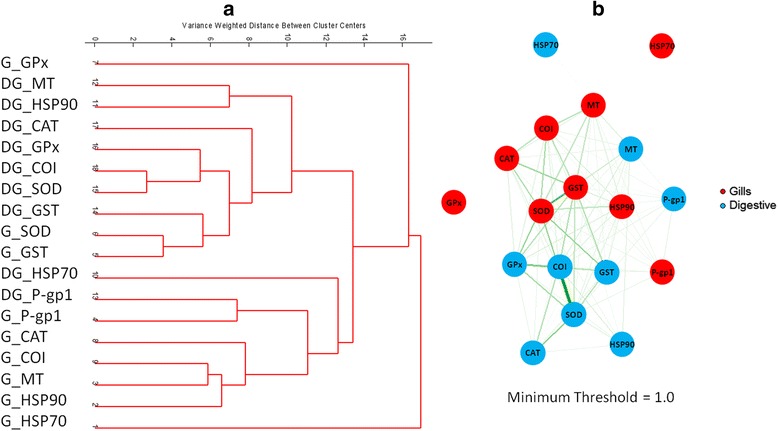
Clustering analysis. **a)** Dendrogram showing the relations between genes, and **b)** Gene network corresponding based on the matrix of distances used to build the dendrogram (width of the lines is inversely proportional to the distance).

In dendrogram of Figure 3, genes GPx and HSP70 from gills had a totally different behaviour since they did not show any similarity with other genes. Apart from them, two main groups of genes were distinguished which could be assigned to either of the two investigated tissues. In the upper part of the dendrogram, genes were related to digestive gland tissue whereas those in its lower part were related to gills tissue. It is worth to highlight that SOD and GST genes from gills were located in the branch of the dendrogram associated with the digestive gland, which is probably due to the similarity between the gene expression variations caused by heavy metals in digestive glands (specially GST and, in a minor extent, SOD, COI and GPx), and that of SOD and GST in gills. It is also important to point out that genes of digestive glands that appeared in the branch of the dendrogram mostly associated to gills are HSP70 (which was the gene with the most different behaviour) and P-gp1 (which formed a cluster with the same gene from gills).

As in the previous case, Figure 3b shows the gene network diagram built from the reciprocal of the distances obtained between genes in the hierarchical clustering approach described above. This representation showed some advantages with respect to previous dendrogram approach. For instance, relationships between genes can be seen in a more intuitive and visual way allowing an easier association of the genes from different clusters.

In this figure, clusters built up by the genes were most strongly correlated in both gills and digestive glands. SOD, COI, GST and CAT formed a group because of their correlation in both tissues. Relations between genes in both tissues can be observed at the edges that connect nodes of gills and digestive glands. For instance, the relationship between SOD genes in gills and GPx genes in digestive glands were stronger than other relations observed for genes of the same tissues. Finally, HSP70 and GPx in gills and HSP70 in digestive glands were confirmed not to have any correlation with the other investigated genes.

When interpretation of these results using non-supervised data analysis methods is complemented with the information available in the literature, it is observed that genes with stronger correlations in both gills and digestive glands (SOD, CAT, CAI, GST, and GPx) are known to be related to the oxidative metabolism which is significantly affected by heavy metals exposure.

### Principal component analysis results

Principal component analysis (PCA) is used to extract relevant information about samples clustering and effects of metal exposure treatments. The information given by PCA analysis will be compared with that obtained through graphical means in the previous section. First principal component, PC1, already explained 45.1% of the observed variance of the experimental data. The rest of principal components explained a lower amount of experimental variance: PC2 – 19.5%, PC3 – 10.5% and PC4 – 7.7%. From PC5, the amount of explained variance was lower than 5%.

**Figure 4:**
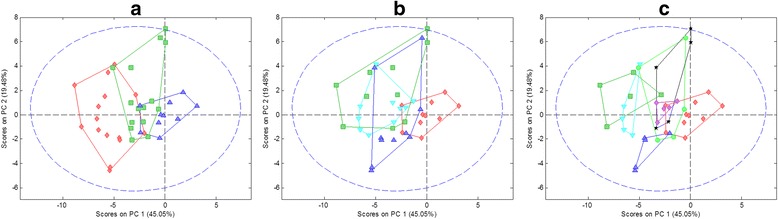
Principal component analysis: scores plots. Scores plots (PC2 *vs.* PC1) with legend based on **a)** exposure time: control samples – blue triangles, 1-day treated samples – red diamonds and 7-days treated samples – green squares, **b)** treatment type: control samples – red diamonds, Cu treated samples – green squares, Cd treated samples – blue up-triangles, Hg treated samples – cyan down-triangles, and **c)** combination of exposure time and treatment type: Control samples – red diamonds, Cu and 1-day samples – green squares, Cd and 1-day samples – blue up-triangles, Hg and 1-day samples – cyan down-triangles, Cu and 7-days samples – black stars, Cd and 7-days samples – green circles and Hg and 7-days samples – violet diamonds.

Figure 4 shows the scatter scores plots that related the first two principal components with the samples labelled according to exposure time, treatment, and the combination of exposure time and treatment. Additional file 1 Figure S1 in shows the scatter plots considering the first three components. In these plots, control samples were close to the origin due to mean-subtraction pre-treatment of control samples prior to data analysis.

From the analysis of these plots, it can be observed the influence of the exposure time on PC1 (Figures 4a). Samples with one-day exposure time showed high negative values while samples with one-week exposure time appeared closer to the origin of coordinates. In Figures 4b separation of samples according to heavy metal treatment is presented. In PC2 *vs.* PC1 plots, Cu – Hg – Cd could be differentiated along PC2 (samples related with each applied metal could be seen). If PC3 was considered, a cluster of Cd treated samples was separated from the rest of samples.

Considering both treatment effects simultaneously (metal and exposure time), the exposure time separated each metal cluster into two sub-clusters within each type of metal group. For instance, in the PC2 *vs.* PC1 scatter plot (Figure 4c) the three considered metals could be clearly distinguished when considering one-day samples whereas this differentiation was not so obvious when considering one-week samples.

**Figure 5:**
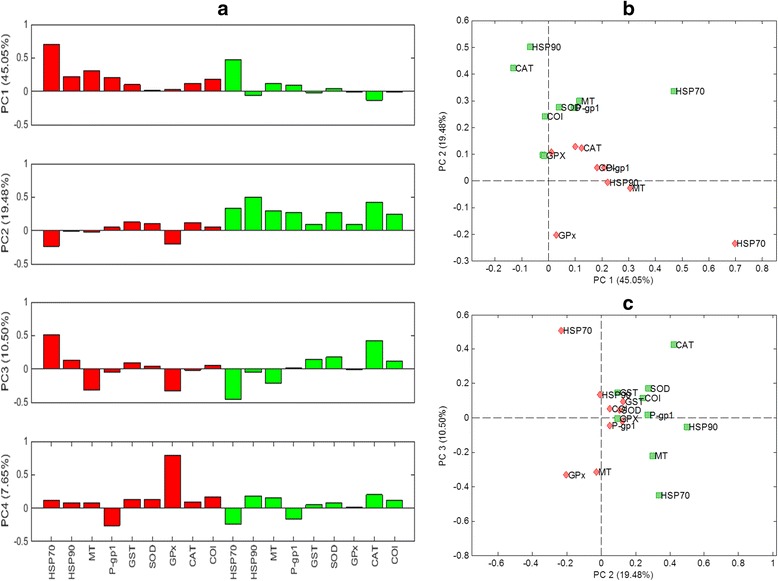
Principal component analysis: loadings plots. **a)** Individual bar diagrams for PC1, PC2, PC3 and PC4 (gills: red bars, digestive glands: green bars), **b)** PC2 *vs.* PC1 scatter plots and **c)** PC3 *vs.* PC2 scatter plots (in b) and c) gills genes: red diamonds and digestive glands genes: green squares).

In Figure 5, loading plots identified what genes were the most related with each principal component. Differentiation between gills and digestive gland genes was mainly displayed by the second principal component. Genes associated with gills at PC2 had significantly lower values than genes linked to digestive glands (Figure 5a-2^nd^ plot, and Figures 5b and c). Therefore, two types of gene clusters were differentiated based on the tissue from which they were obtained.

In Figure 5a, PC1 was mainly influenced by the HSP70 gene from both tissues. The main effect observed on PC2 was showing the separation of genes by tissue as discussed above. Genes that exhibited a higher contribution on PC2 were HSP90 and CAT in case of digestive glands, and GPx and HSP70 in the case of gills. SOD, CAT and COI (and in a lesser extent GST and GPx) showed a significant contribution only for genes of digestive glands in PC2 (Figure 5a). However, all genes behaved similarly in both tissues. This could be checked in the scatter loadings plots where genes were close to each other for each tissue and, in addition, they were in a closer region when both tissues were considered.

Summarizing PCA results, PC1 could be related with the exposure time of samples. Among genes that mostly contributed to PC1, the HSP70 gene could be identified as the one showing higher positive score values (in both tissues). This indicates that this gene allowed differentiating samples according to accumulative toxic effects across time. On the same manner, the diagonal trend in PC2 *vs.* PC1 scores plot enabled the differentiation among samples as a function of heavy metal treatment.

From gene loadings, HSP90 and CAT of the digestive glands were correlated with copper treated samples while HSP70 of gills was mainly correlated to cadmium treated samples. The determination of the genes most correlated with the Hg treated samples was not straightforward due to their closeness to the origin.

From PC2 loadings plots differentiation of genes according to tissue type was possible. Samples behaved differently and only in the case of cadmium treated samples, a cluster was identified. Cd treated samples with one-day of exposure time showed negative values of PC2 which could be related to gills genes expression, while Cd treated samples with one-week of exposure time showed positive PC2 values which could be linked with digestive gland genes.

### ANOVA simultaneous component analysis results

**Figure 6:**
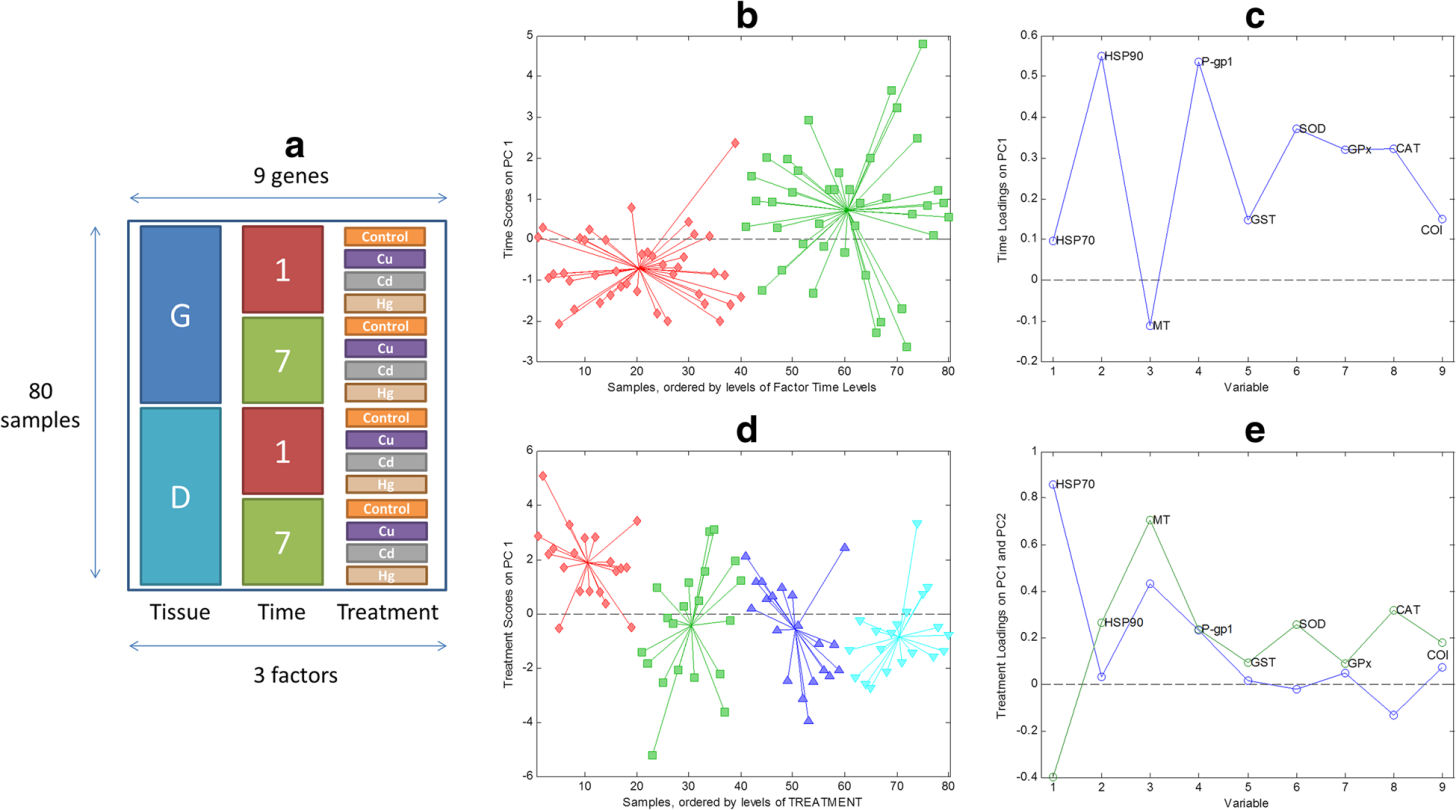
ASCA analysis **a)** Scheme of the matrix for ASCA analysis. ASCA results: Effect of exposure time: **b)** PC1 SCA scores values (Legend: 1-day: red diamonds, 7-days: green squares) and **c)** PC1 SCA loadings plot (Legend: Loadings PC1: blue line). Effect of treatment: **d)** PC2 *vs.* PC1 SCA scores plot (Legend: Control: red diamonds, Cu: green squares, Cd: blue up-triangles, Hg: cyan down-triangles), and **e)** PC2 *vs.* PC1 SCA loadings of metal treatment factor matrix (Legend: Loadings PC1: blue line, Loadings PC2: green line).

For ANOVA simultaneous component analysis (ASCA), the data matrix was rearranged as can be seen in Figure 6a. Note that in ASCA, ANOVA is applied to multivariate gene responses. Three experimental factors were considered in the ASCA analysis: tissues (gills or digestive gland), exposure time (one or seven days) and type of treatment (control, cadmium, mercury or copper).

Statistical significance of these factors was estimated by using a permutation test approach. In this work, the number of permutations was set to 100000. Only individual effects of exposure time **X**_**e**_ (*p*_time_ = 0.00505) and treatment **X**_**t**_ (*p*_treatment_ = 0.00003) were significant, and allow rejecting the null hypothesis H_0_. In all the other cases, the null hypothesis (H_1_) was accepted (there was no significant effect of the considered factor or interaction). The triple interaction treatment-tissue-time and the double interactions tissue-time, tissue-treatment, and time-treatment provided *p*-values rather close to 1. Finally, individual effects of tissue (**X**_**T**_) was not considered statistically significant (*p*_tissue_ = 0.4540).

Figure 6 shows the representation of scores and loadings matrices related to the individual effect of exposure time and metal treatment. For exposure time individual factor matrix, only one principal component was needed to explain most of the variance. Figure 6b shows the projected scores where one-day and one-week samples can be distinguished. Loadings plot (Figure 6c) displays the high influence of the HSP90, MT and P-gp1 genes in the two first principal components.

For metal treatment, three principal components were needed. Effects in projected scores (Figure 6d) and loadings (Figure 6e) can be interpreted in a similar way than that for the PCA analysis. Treated samples could be clearly distinguished from the control samples on the first principal component whereas the second component allowed grouping the different metal treatments with some overlapping. In the case of the loadings plot, effects of gene HSP70 and MT were distinguished. These results also were concordant with those obtained in the PCA analysis. (Figure 6d).

### Partial least squares discriminant analysis results

Partial least squares discriminant analysis models were used to identify the more discriminant variables among different type of samples considering exposure time and metal treatment as possible factors.

**Figure 7:**
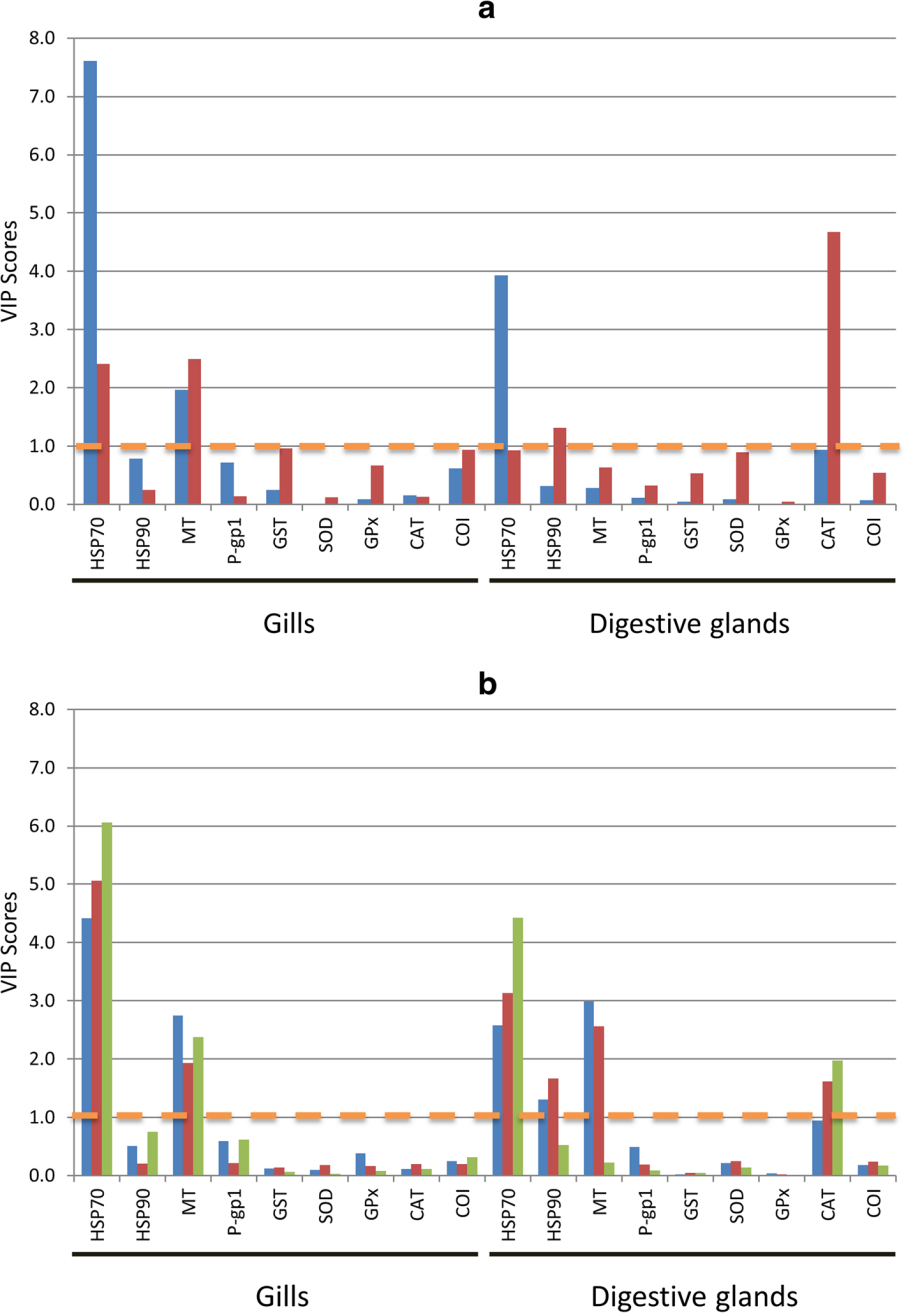
PLS-DA results. Bar diagram showing the VIP Scores of the PLS-DA model based on **a)** exposure time (1-day: blue, 1-week: red), and **b)** metal treatment type (Cu: blue, Cd: red, Hg: green). VIP scores threshold for selecting variables was set to 1.

In the case of exposure time, two PLS-DA models were built up: one for the discrimination of one-day samples and the other for the discrimination of one-week samples. In both cases, discrimination between samples classes achieved by the PLS-DA model was good as can be seen in the obtained sensitivity, specificity, and accuracy parameters (see Table 1). When VIP scores of the each PLS-DA model were considered (Figure 7a), the most relevant genes, for discriminating between samples, were obtained. In the case of one-day exposure time, the HSP70 gene (both in gills and digestive gland tissues) was the more discriminating variable. On a minor extent, MT gene of gills also allowed discriminating one-day samples. In the case of one-week exposure time, CAT (digestive gland) gene was the most relevant together with MT, HSP70, and HSP90, at a lower extent.

**Table 1:** **PLS-DA quality parameters**

**Factor**	**Class**	**Sensitivity**	**Specificity**	**Accuracy**
**Time of exposure**	1-day	0.93	0.88	0.91
	1-week	0.86	0.96	0.91
**Metal treatment**	Cu	0.70	0.80	0.77
	Cd	0.80	0.77	0.78
	Hg	0.80	0.67	0.73

Sensitivity = TP/(TP + FN); Specificity = TN/(TN + FP); Accuracy = (TN + TP)/(TN + TP + FN + FP) where TP are true positives, TN are true negatives, FP are false positives, and FN are false negatives.

In the case of samples treated with heavy metals, three PLS-DA models were built up corresponding for each type of treatments (Cu-treated, Cd-treated and Hg-treated samples). Figures of merit of PLS-DA models shown in Table 1 indicated discrimination was acceptable in the three cases with similar results. VIP scores for the three models represented in Figure 7b were rather similar. HSP70 gene (both for gills and digestive glands tissues) was the more discriminant variable in the three cases, probably due to the strong effect of the exposure time discussed above. Apart from this strong influence on HSP70 genes, Cu treated samples were also influenced by MT (gills and digestive glands) and HSP90 (digestive glands) genes. In Cd treated samples, MT gene was the discriminating variable (in both tissues) and, also, in a minor extent, CAT and HSP90 genes. Finally, in Hg treated samples MT (gills) and CAT (digestive glands) genes were the more relevant discriminant variables.

## Discussion

### Phylogenetic analysis of co-regulated genes

**Figure 8:**
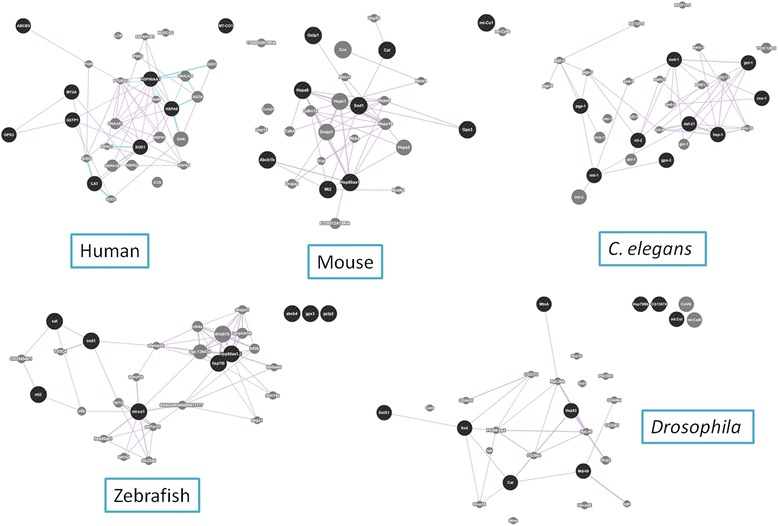
Comparison of gene networks. Gene networks for the genes putatively homologous to the ones analyzed in zebra mussel in Human, Mouse, Zebrafish, *Drosophila* and *C. elegans.* Both the gene expression correlation data and the graphic output were obtained from the GeneMANIA web page (http://genemania.org).

Genetic and regulatory interactions between stress genes in *D. polymorpha* (Figures 2 and 3) were also explored in different model species using the respective putative orthologs (Table 2). While some uncertainties are unavoidable when adscribing orthologs for genes from *D. polymorpha* in other species, some of the co-regulatory interactions shown in Figures 2 and 3 were also seen in phylogenetic distant species (Figure 8).

**Table 2:** **D. polymorpha stress genes’ homologs in reference model species**

***D. polymorpha***		***H. sapiens***		***M. musculus***		***D. rerio***		***C. elegans***		***D. melanogaster***	
Gene name	GB reference	Gene name	GB reference	Gene name	GB reference	Gene name	GB reference	Gene name	GB reference	Gene name	GB reference
MT	U67347	MT2A	NC_000016.10	mt2	NC_000074.6	mt2	NC_007129.6	mtl-1	NC_003283.10	MtnA	NT_033777.3
HSP70	EF526096	HSPA8	AAH07276.2	Hspa8	AAI06170.1	hsp70-4	AAH56709.1	HSP-1	NP_503068.1	hsp70Bb	AAW34352.1
HSP90	GU433881	HSP90AA1	NC_000014.9	Hsp90aa1	AAA37868.1	hsp90aa1.2	AAI54424.1	DAF-21	NP_506626.1	Hsp83	AAB46685.1
GST	EF194203	GSTP1	AAC13869.1	GSTP1	NP_038569.1	gstp2	NP_001018349.1	GST-1	NP_499006.1	GstS1	NP_725653.1
SOD	AY377970	SOD1	NP_000445.1	SOD1	NP_035564.1	sod1	NP_571369.1	SOD-1	NP_001021956.1	Sod	NP_476735.1
GPx	DQ459994	GPX3	NP_002075.2	GPX3	AFP27210.1	gpx3	NP_001131027.1	GPX-3	NP_509616.1	CG13074	NP_648835.1
CAT	EF681763	Cat	AAB59522.1	cat	AAA66054.1	cat	AAF89686.1	CTL-2	NP_001022473.1	cat	NP_536731.1
COI	AM749000	Coi	AEH94123.1	CoI	AAX19525.1	coi	AFG23394.1	cco-1	NP_006961.1	mt:CoI	ADG46971.1
P-gp1	AJ506742	ABCB5alpha	AAW31629.1	Abcb1b	NP_035205.1	abcb4	NP_001108055.1	PGP-1	NP_502413.1	Mdr49	NP_001163132.1

**Figure 9:**
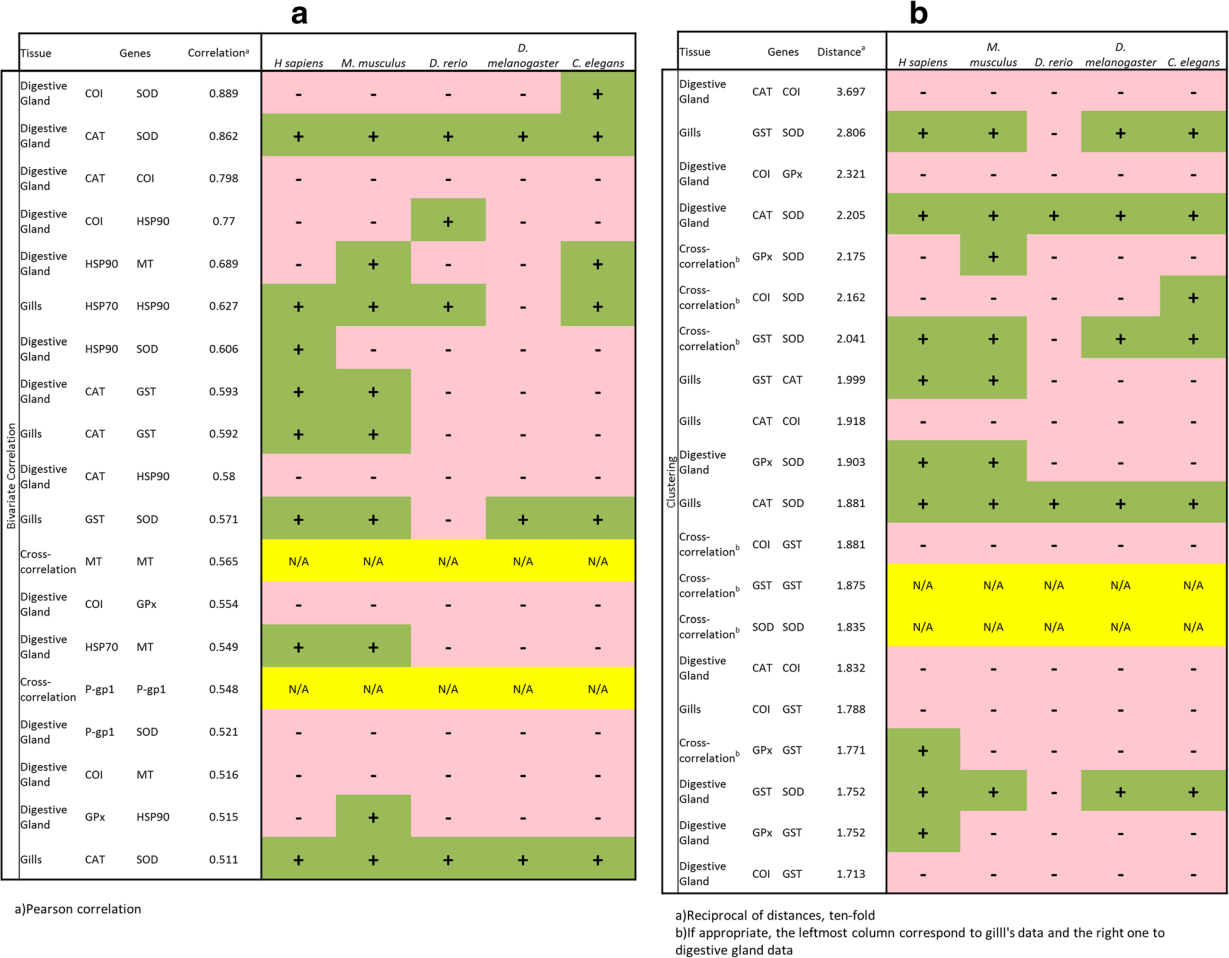
Comparison of the co-expression gene data between species. **a)** bivariate correlations (see Figure 2) between zebra mussel data and the different model species shown in Figure 8. b) clustering analysis (see Figure 3). Only gene pairs with 2 the highest correlations and/or lowest distance values are considered. Distance values are represented as reciprocals in **b)**. Green cells with plus signals indicate correlations also observed for the corresponding model species; pink cells with minus signals indicate otherwise. N/A in a yellow background indicates non-applicable interspecies correlations.

For example, GST-SOD and GST-CAT co-expression seem to be quasi-universal, at least within Metazoans, as well as the interactions between HSP90 and HSP70 (Figure 9). Conversely, co-expression between MT and HSP90 was only found in two species (mouse and *C. elegans*), whereas COI and P-gp1 genetic interaction with other stress genes was rarely (if ever) observed in the other species (Figure 9). Given the wide evolutionary gap between *D. polymorpha* and vertebrates or *D. melanogaster* and *C. elegans*, the existence of common co-expression patterns indicates that the correlation analyses were able to define deeply rooted regulatory networks among Metazoans. GST, SOD, and CAT are part of the cellular defence mechanism against oxidative stress, although they act at very different levels [25,26]. Similarly, HSP90 and HSP70 share the heat-shock responsive element (also found in some metallothionein genes, [27,25]), so its co-expression may well be mediated by this particular regulatory network. The mechanisms for the co-expression of COI (an essential mitochondrial component required for cellular respiration) and the components of the oxidative stress cluster seems to be a unique of *Dreissena*, and may indicate a subjacent defence mechanism not characterized yet. The same apply to the correlation between Pgp-1 and SOD, only observed in gills (Figure 9). The possible meaning of these observations will be only understood as our knowledge of the defence mechanisms in molluscs increases. Our results suggest some mechanism(s) linking the presence of stress agents (in this case, heavy metals) to at least four levels of cellular defence: 1) Out flux of the exogenous agent (Pgp-1); 2) Chelation and neutralization of divalent metals (MT); 3) Heat-shock response (HSP70 and HSP90), probably related to the presence of denatured proteins; and 4) Oxidative stress defence (GST, SOD, CAT). The ASCA analysis suggests a temporal gradation of these mechanisms, being HSP90 and Pgp-1 expression more related to the early response (one-day, Figure 6b-6c), and MT and HSP70 associated to the chronic exposure (Figure 6b-6c). At this point, it should be considered that longer exposure times imply two independent and somewhat contradictory mechanisms. In the first place, tissue damage may accumulate over time, increasing the toxic effects. At the same time, acclimation processes occur, by which cells (and tissues) compensate the presence of the toxicant and reduce its effective toxicity. These two opposite effects may well be the reason for the negative, quasi-linear correlation of PC1 and PC2 scores in Figure 5b. The fact that the correlation analyses were able to identify these different defence modules and following a co-expression pattern similar to, or at least compatible with, those already known for reference model species demonstrate the utility of these statistical methods to explore regulatory networks in species, like *D. polymorpha*, for which very little genetic information is available.

### Agent- and tissue-specificity of the stress response

While a direct evaluation of the severity of the toxic effects is not possible with the current data, clustering of the different samples shows that mercury-treated samples were closer to controls than Cd- or Cu-treated ones, suggesting that these two heavy metals were more toxic to *D. polymorpha* than mercury. This conclusion was also drawn from the preliminary analysis of this data [22] as well as from a transcriptomic analysis of *D. polymorpha* populations along a pollution gradient in the Ebro River (Spain, [23]). The pattern of response seemed to differ for the two analyzed tissues as shown in Figures 2 and 3. However, expression of two genes (Pgp-1 and MT) appeared to be highly coordinated in both tissues (Figures 2 and 3, see also [22]). It is important to note that these two genes directly interact with the toxic agent (extruding it out of the cell in the first case, and chelating it in the second case), whereas the other mechanisms are compensating potentially deleterious alterations in the cell components (oxidation, denatured proteins). Therefore, it is not unrealistic to think that Pgp-1 and MT expression reflected the effective concentration of the metals in both tissues (bound to be relatively similar), whereas expression of the other genes would depend upon the extent of these internal damages, which very likely differ for both cell types.

## Conclusions

In this work, the application of different chemometric methods allowed the extraction of relevant information from qRT-PCR data. Results from different methods appeared to be complementary focusing on various data features. Information provided by gene network diagrams can make rather easy the interpretation of the possible correlations between investigated genes.

Chemometric results showed that genes were clustered according to the type of tissue, and separation of samples was achieved according to their time evolution (one-day versus one-week treatment) and heavy metal treatment. It is remarkable the conservation of at least some of the regulatory networks within Metazoans, and the ability of the presented method to define these genetic interactions using only a limited number of experiments and conditions in species, such as *D. polymorpha*, for which very little genetic information is available.

## Methods

### Data studied

A short introduction about qRT-PCR is presented below. qRT-PCR measures the fluorescence of the PCR reaction products during a cycle threshold (C_p_). Above this threshold value, the fluorescence of the samples is considered to be above the background contribution [5]. This C_p_ value is related to the initial concentration of RNA that allows its absolute quantification, according to Equation 1:

1 $$ {\mathrm{C}}_{\mathrm{p}} = -\mathrm{k}\  \log\ \left[\mathrm{R}\mathrm{N}\mathrm{A}\right] $$

However, relative quantification is usually performed by calculating the difference between Cp values of the considered gene and of the housekeeping (control) DNA sequence [28,29]:

2 $$ \Delta {\mathrm{C}}_{\mathrm{p}} = {\mathrm{C}}_{\mathrm{p},\mathrm{r}\mathrm{e}\mathrm{f}}\hbox{--}\ {\mathrm{C}}_{\mathrm{p},\mathrm{sample}} $$

In this work, qRT-PCR measurements allowed building up a data matrix of 120 rows (samples) and 24 columns (variables). These 120 rows included 3 technical replicates of 20 different mussel samples (5 control samples, 5 treated samples with Cd, 5 treated samples with Cu and 5 treated samples with Hg) measured at two different treatment times (1 day and 7 days after metal exposure), respectively. For each sample, 24 measurements were obtained corresponding to the expression responses of 12 selected genes (S3, EF1, BAct, MT, HSP70, HSP90, GST, SOD, GPx, CAT, COI, and P-gp1, details in Additional file 2 Table S1) from gills and digestive glands of the same individuals. More details about the experimental procedure related to data acquisition can be found at Navarro et al. [22].

### Data preparation and pre-treatment

Experimental qRT-PCR data presented some initial problems that hampered their direct exploration. First, since approximately 8% of data values were missing, the average of the three technical replicates was calculated to obtain a value for each combination sample-gene. This strategy gave a total number of measurements reduced to 40. Next, for relative quantization estimations, one reference gene should be selected among those that explain minimum variance for both tissue types (gills and digestive gland). In both cases, the best housekeeping gene was S3. Other genes that showed minor variations across samples were EF1 or BAct, but they were not selected as a reference gene and therefore discarded for further analysis. Final size of experimental data matrix was 40 rows (20 samples after one day and 20 samples after one week of exposure) and 18 columns (9 genes for gills and 9 genes for digestive gland). A schematic representation of the dataset built up is shown in Additional file 1: Figure S1.

For ASCA analysis, this data matrix was rearranged with the goal of obtaining more information from the study. Gene measurements from different tissues were considered as different samples generating a final matrix of 80 rows (40 samples for gills and 40 samples for digestive glands) and 9 columns (corresponding to the 9 different genes)

Finally, data were mean centred prior to chemometric analysis. Figure 1 shows experimental data after S3 reference gene subtraction before (Figure 1b) and after mean centring pre-treatment (Figure 1c). Moreover, mean responses of control samples for each tissue were subtracted for PCA and PLS-DA analysis.

### Data mining and phylogenetic comparative analyses

Putative orthologues for the nine stress genes analysed in *D. polymorpha* (MT, HSP70, HSP90, GST, SOD, GPx, CAT, COI, and P-gp1) in five reference model species: human, *Homo sapiens*; mouse, *Mus musculus*; zebrafish, *Danio rerio*; the fruit fly *Drosophila melanogaster*; and the nematode *Caenorhabditis elegans*, were identified by the BLAST algorithm at NCBI server, (http://www.ncbi.nlm.nih.gov/blast/Blast.cgi) using the complete sequences of the corresponding *D. polymorpha* genes (Listed in Table 2). Genetic and regulatory interactions between the different genes in each model species were explored using the GeneMANIA web page (http://genemania.org) [30].

#### Data analysis methods

##### Gene inspection, clustering and networking

Initially, the gene correlation data matrix was investigated. Two visualization tools were used in order to extract the main relevant information from this correlation matrix in a natural and intuitive manner. On one side, the heat map graphical representation of the correlation matrix was considered. An ellipse-based color codification was used to indicate if correlation values are positive (blue) or negative (red). Moreover, the length of the minor axis of the ellipse indicates the strength of the correlation (the shorter the minor axis is, the stronger correlation exists) [31]. On the other side, the *qgraph* tool of the R environment [32] was used to visualize gene interactions in a network. This tool allows for data representation in a simple network where each variable (gene) is a node, and each edge shows the correlation between two genes. The thickness of the line on these edges is related to the size of this correlation.

Secondly, non-supervised hierarchical clustering analysis (HCA) was used to display correlations between genes. Cluster analysis is used to classify objects, characterized by the values of a set of variables, into clusters or groups [33]; in such a way that one object within a cluster is more closely related to one object of the same cluster than to another object assigned to a different cluster. In order to build up these clusters or groups, a measurement of the similarity or distance between the various objects is needed. Examples of possible distance measures are the Euclidean, City block or Mahalanobis distances. Additionally, there are several agglomerative linkage cluster methods such as Nearest Neighbor, Furthest Neighbor, Centroid, Median or Ward’s Method [24]. In this work, the Euclidean Distance and the agglomerative Ward’s method have been selected.

Two different display outputs can be obtained using this clustering process, the dendrogram, which is a tree-like diagram illustrating HCA clusters and the matrix of gene distances values [33]. Distances matrix can be used as an initial basis for visual representation of the gene network, as the correlation matrix. Similarly, gene network also displays the interactions and correlations between different genes.

### Principal component analysis

Principal component analysis (PCA) is based on the fulfilment of a bilinear model that decomposes experimental data in the product of two factor matrices related respectively to sample (rows) and variable (columns) contributions, using a minimum number of components to explain most of the data variance [34]:

3 $$ \mathbf{X}=\mathbf{T}\ {\mathbf{P}}^{\mathbf{T}}+\mathbf{E} $$

In this Equation PCA, **X** is the experimental data matrix of size *m* samples (rows) and *n* genes (variables, columns), **T** is the factor matrix related to sample contributions (usually known as scores) of size *m* number of samples and *Ns* number of principal components selected in the analysis, **P**^**T**^ corresponds to the matrix related to the gene contributions (to the variables, usually known as loadings) of size *Ns* number of principal components and *n* number of genes. Finally, matrix **E** (of size *m* samples and *n* genes) contains the variance not explained by the bilinear model for the considered number of principal components, *Ns*. Every one of these *Ns* components is characterized by two vector profiles related respectively to the individual samples responses and with its gene expression. Hence, from these two profiles associated with each principal component, biological interpretation of each one of these components can be inferred [35]. In summary, PCA has been used to identify relationships between treated samples according to their gene expression and, also, to investigate possible relations between genes.

### ANOVA simultaneous component analysis

There are different data analysis approaches that combine the power of ANOVA with that of PCA to identify and separate variance sources [36,24]. In this work, the selected approach has been the ANOVA simultaneous component analysis (ASCA) method [37].

In ASCA, SCA (similar to previously described PCA) is applied separately to each effect matrix and to all possible interaction matrices. First, the data matrix **X** is split into effect matrices containing the level averages for each factor and interaction matrices that describes the interaction between the considered factors [38]. In the case of the three factors considered in this work (tissue, exposure time and metal treatment), this is written as:

4 $$ \mathbf{X}=\overline{\mathbf{X}}+{\mathbf{X}}_{\mathbf{T}}+{\mathbf{X}}_{\mathbf{e}}+{\mathbf{X}}_{\mathbf{t}}+{\mathbf{X}}_{\mathbf{T}\mathbf{e}}+{\mathbf{X}}_{\mathbf{T}\mathbf{t}}+{\mathbf{X}}_{\mathbf{e}\mathbf{t}}+{\mathbf{X}}_{\mathbf{T}\mathbf{e}\mathbf{t}} + \mathbf{E}=1{\mathbf{m}}^{\mathbf{T}}+{\mathbf{T}}_{\mathbf{T}}{\mathbf{P}}_{\mathbf{T}}^{\mathbf{T}}+{\mathbf{T}}_{\mathbf{e}}{\mathbf{P}}_{\mathbf{e}}^{\mathbf{T}}+{\mathbf{T}}_{\mathbf{t}}{\mathbf{P}}_{\mathbf{t}}^{\mathbf{T}}+{\mathbf{T}}_{\mathbf{T}\mathbf{e}}{\mathbf{P}}_{\mathbf{T}\mathbf{e}}^{\mathbf{T}}+{\mathbf{T}}_{\mathbf{T}\mathbf{t}}{\mathbf{P}}_{\mathbf{T}\mathbf{t}}^{\mathbf{T}}+{\mathbf{T}}_{\mathbf{e}\mathbf{t}}{\mathbf{P}}_{\mathbf{e}\mathbf{t}}^{\mathbf{T}}+{\mathbf{T}}_{\mathbf{T}\mathbf{e}\mathbf{t}}{\mathbf{P}}_{\mathbf{T}\mathbf{e}\mathbf{t}}^{\mathbf{T}}+\mathbf{E} $$

In this equation, **X** is the experimental data matrix of i rows and j variables, $\overline{\mathbf{X}}$ is the grand mean data matrix, **X**_**T**_ is the effect of tissue factor (gills or digestive glands), **X**_**e**_ is the effect of exposure time factor (one-day or one-week), **X**_**t**_ is the effect of metal treatment factor (control samples, copper, cadmium or mercury treated samples), **X**_**Te**_ is the interaction of tissue and exposure time factors, **X**_**Tt**_ is the interaction of tissue and metal treatment factors, **X**_**et**_ is the interaction of exposure time, and metal treatment factors and **X**_**Tet**_ is the global interaction of tissue, exposure time and metal treatment factors. In addition, 1 is a vector of ones of *i* rows and *m* is a vector of the overall means of the experimental matrix (*j* rows). For each submodel of factors or interactions, there are the associated component scores (**T**_**T**_, **T**_**e**_, **T**_**t**_, **T**_**Te**_, **T**_**Tt**_, **T**_**et**_ and **T**_**Tet**_) and component loadings (${\mathbf{P}}_{\mathbf{T}}^{\mathbf{T}}$, ${\mathbf{P}}_{\mathbf{e}}^{\mathbf{T}}$, ${\mathbf{P}}_{\mathbf{t}}^{\mathbf{T}}$, ${\mathbf{P}}_{\mathbf{T}\mathbf{e}}^{\mathbf{T}}$, ${\mathbf{P}}_{\mathbf{T}\mathbf{t}}^{\mathbf{T}}$, ${\mathbf{P}}_{\mathbf{et}}^{\mathbf{T}}$ and ${\mathbf{P}}_{\mathbf{T}\mathbf{et}}^{\mathbf{T}}$). Finally, **E** corresponds to the residuals of all submodels of the global ASCA model: **E** = **E**_**T**_ + **E**_**e**_ + **E**_**t**_ + **E**_**Te**_ + **E**_**Tt**_ + **E**_**et**_ + **E**_**Tet**_.

Since different PCA models are fitted to each effect matrix that contains the averages of the measurements with the same factor settings, they do not represent the natural variation of the data [37]. This fact causes that the ASCA scores do not show the variation between replicates for each combination of factor levels. Hence, the estimation of the replicates variation in the PCA subspace of a factor is given by Equation 5:

5 $$ {\mathbf{Y}}_{\mathbf{k}}=\left({\mathbf{X}}_{\mathbf{k}}+\mathbf{E}\right)\mathbf{P}={\mathbf{T}}_{\mathbf{k}}+\mathbf{E}{\mathbf{P}}_{\mathbf{k}} $$

The projection for each factor **Y**_**k**_ describes the variation among replicates in the principal component subspace of the considered factor *k*. Effect matrix (**X**_**k**_), residual matrix (**E**) and loadings (**P**_**k**_) are used to obtain the projection matrix.

The assessment of the statistically significance of the effects of all factors and of their interactions is checked under the null hypothesis H_0_ of no experimental effect (no difference between the level averages of the effect matrices) against the alterative hypothesis of the presence of an experimental effect with a *p* confidence level. The estimation of this *p*-value is obtained by a permutation test, in which the original data matrix is permuted a number of times and the sum of the squares (SSQ) of the *k* effect matrix is recalculated (i.e. 100000 permutations) [39]:

6 $$ \mathrm{S}\mathrm{S}\mathrm{Q} = {\displaystyle \sum_{\mathrm{i}}^{\mathrm{N}}}{\displaystyle \sum_{\mathrm{m}}^2}{\left({\mathrm{T}}_{\mathrm{k}}\right)}_{\mathrm{i},\mathrm{m}}^2 $$

where the first summation (*i*) correspond to the total number of samples (*N*) and the second summation (*m*) to the considered principal component (maximum 2). The probability *p*-value is estimated from the number of permutations that give an SSQ value that is larger than the SSQ obtained for the experimental data.

### Partial least squares discriminant analysis

Partial least squares discriminant analysis (PLS-DA) is the application of PLS method for discrimination purposes [40]. In PLS-DA, the dependent variable (to be predicted), **Y,** is a vector or matrix that codifies the pertinence or not of a given sample to a particular sample class or type. In this method, **X** contains the input information about the gene expression samples response (qRT-PCR data) after the different considered treatments (exposure time and metal type). Internal cross-validation by random subsets of the samples was used to evaluate the reliability of the obtained model. The PLS method constructs a set of loading weights (or weights) **W**, which give the relationships between **X** and **Y** during the regression process. Each one of the **w**_**i**_ vectors is orthogonal from each other and characterize the PLS component direction in the **X**-space, which is optimally correlated with the variation in **Y** [41,42].

From PLS weight vectors, Variable Importance on Projection (VIP) can be calculated to facilitate feature selection. VIP values provide a score value for each variable and rank them according to their significance in the projection used by the PLS model [43,44]. In this way, the higher the VIP score of a particular variable (usually a threshold value of one is used) is the more importance of this variable for the sample discrimination. VIP scores for a certain variable, *j*, are defined as:

7 $$ VI{P}_j=\sqrt{m\raisebox{1ex}{${\displaystyle {\sum}_{k=1}^p}{b}_k^2{w}_{jk}^2$}\!\left/ \!\raisebox{-1ex}{${\displaystyle {\sum}_{k=1}^p}{b}_k^2$}\right.} $$

where *m* corresponds to the total number of variables, *p* is the number of latent variables, *w*_*jk*_ is the *j*-th element of vector *w*_*k*_ and *b*_*k*_ is the regression weight for the *k*-th latent variable.

In this work, PLS-DA has been applied to discriminate samples according to two types of factors: exposure time (with classes: one-day exposure time and one-week exposure time) and heavy metal treatment type (with classes: copper, cadmium and mercury). PLS-DA results provide information about which are the most useful variables for the discrimination between the considered classes, knowledge that can be deduced from the VIP scores.

### Software used

Data pretreatment, hierarchical clustering, PCA, PLS-DA and ASCA analysis have been carried out using the Eigenvector PLS Toolbox (version 7.8.2) for the MATLAB® environment (2013b Release). Correlation Maps with information based on elliptical shapes (corrplot/plotcorr) [31] and Gene Network Maps (qgraph) [32] have been generated using appropriate packages from R environment.

## Additional files

Additional file 1: Figure S1. PCA analysis considering 3 components. Principal Components Analysis scores plots (PC2 *vs.* PC1, PC3 *vs.* PC1 and PC3 *vs.* PC2) with legend based on time of exposure (control samples – blue triangles, 1-day treated samples – red diamonds and 7-days treated samples – green squares): a) PC2 *vs.* PC1, b) PC3 *vs.* PC1, and c) PC3 *vs.* PC2, treatment type (control samples – red diamonds, Cu treated samples – green squares, Cd treated samples – blue up-triangles, Hg treated samples – cyan down-triangles): d) PC2 *vs.* PC1, e) PC3 *vs.* PC1and f) PC3 *vs.* PC2, and combination of exposure time and treatment type (Control samples – red diamonds, Cu and 1-day samples – green squares, Cd and 1-day samples – blue up-triangles, Hg and 1-day samples – cyan down-triangles, Cu and 7-days samples – black stars, Cd and 7-days samples – green circles and Hg and 7-days samples – violet diamonds): g) PC2 *vs.* PC1, h) PC3 *vs.* PC1, and i) PC3 *vs.* PC2. Additional file 2: Table S1. Summary of genes used in this work. Additional file 3: Figure S2. Schematic representation of the analyzed qRT-PCR data matrix.
