# Supplementary figures and images for: qRT-PCR evaluation of the transcriptional response of zebra mussel to heavy metals
Source: BMC Genomics. 2015 May 6;16(1):354. doi: 10.1186/s12864-015-1567-4 (PMC4422313; doi:10.1186/s12864-015-1567-4)

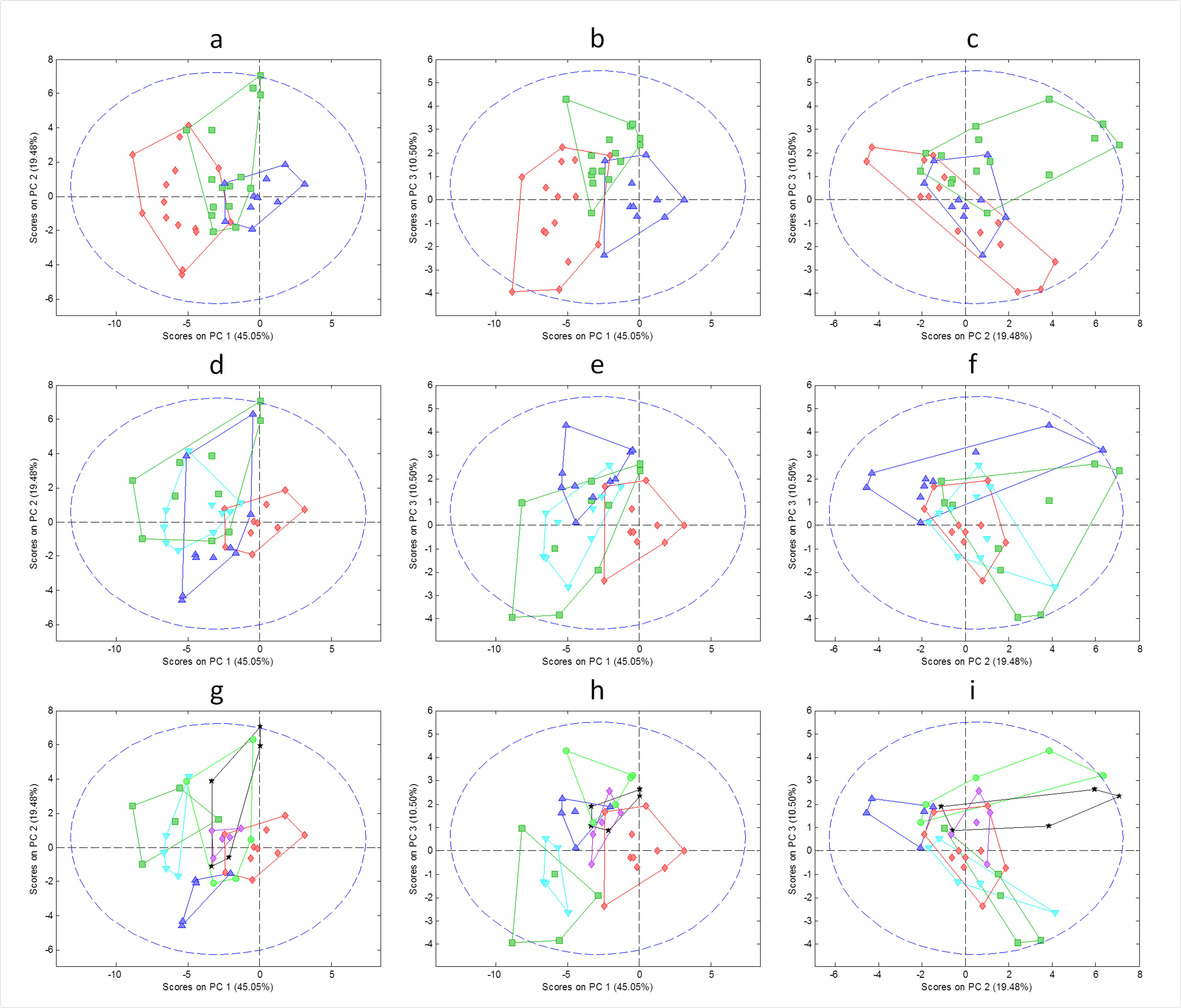

Supplement: Additional file 1: Figure S1. — PCA analysis considering 3 components. Principal Components Analysis scores plots (PC2 vs. PC1, PC3 vs. PC1 and PC3 vs. PC2) with legend based on time of exposure (control samples – blue triangles, 1-day treated samples – red diamonds and 7-days treated samples – green squares): a) PC2 vs. PC1, b) PC3 vs. PC1, and c) PC3 vs. PC2, treatment type (control samples – red diamonds, Cu treated samples – green squares, Cd treated samples – blue up-triangles, Hg treated samples – cyan down-triangles): d) PC2 vs. PC1, e) PC3 vs. PC1and f) PC3 vs. PC2, and combination of exposure time and treatment type (Control samples – red diamonds, Cu and 1-day samples – green squares, Cd and 1-day samples – blue up-triangles, Hg and 1-day samples – cyan down-triangles, Cu and 7-days samples – black stars, Cd and 7-days samples – green circles and Hg and 7-days samples – violet diamonds): g) PC2 vs. PC1, h) PC3 vs. PC1, and i) PC3 vs. PC2. [file 12864_2015_1567_MOESM1_ESM.tiff]

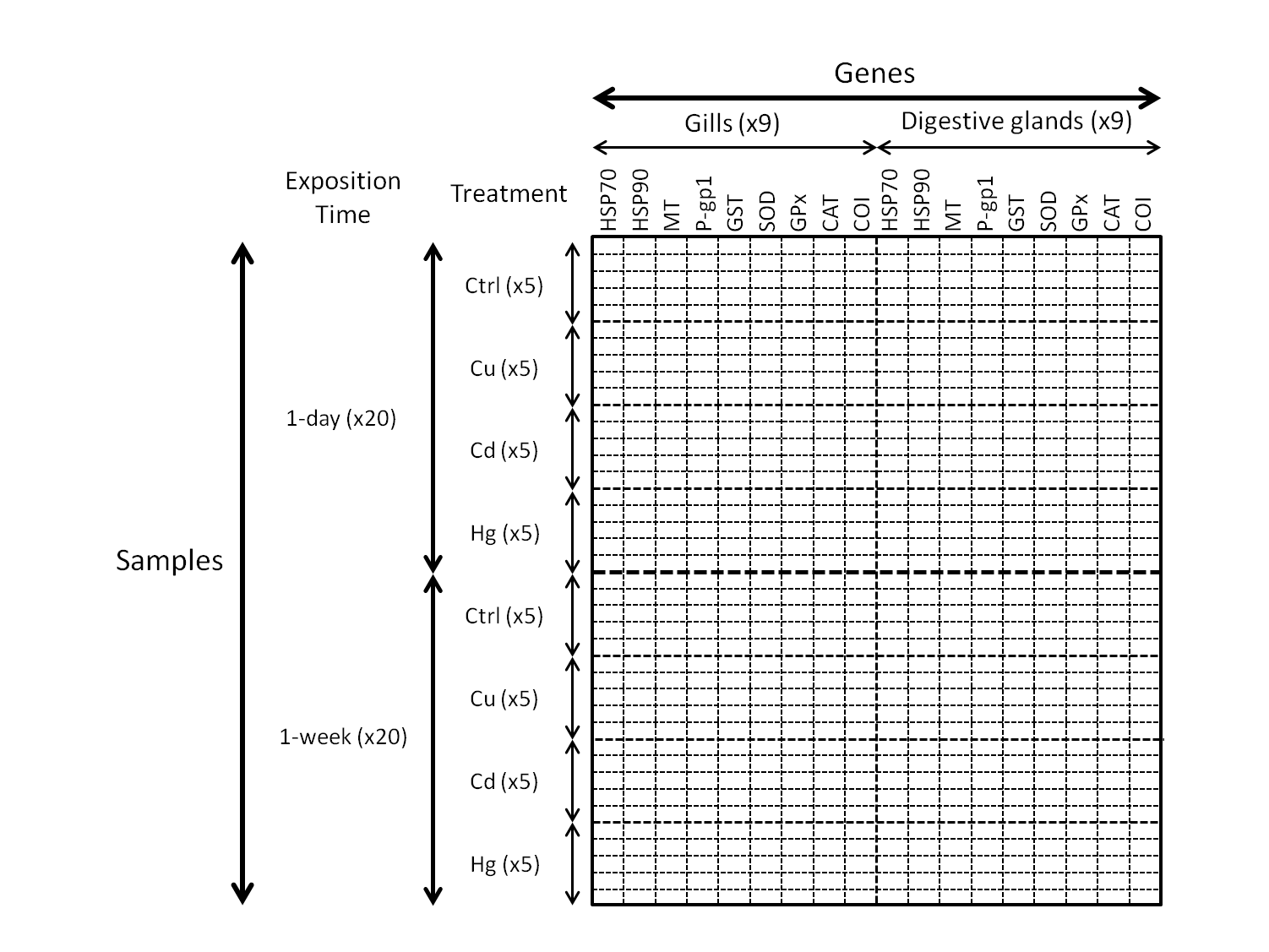

Supplement: Additional file 3: Figure S2. — Schematic representation of the analyzed qRT-PCR data matrix. [file 12864_2015_1567_MOESM3_ESM.tiff]
