# Supplementary material for: qRT-PCR evaluation of the transcriptional response of zebra mussel to heavy metals
Source: BMC Genomics. 2015 May 6;16(1):354. doi: 10.1186/s12864-015-1567-4 (PMC4422313; doi:10.1186/s12864-015-1567-4)
Supplement: Additional file 2: Table S1. — Summary of genes used in this work. [file 12864_2015_1567_MOESM2_ESM.docx]

## Additional file 2 – Table S1: Summary of genes used in this work

| Gene Name | Short Name | Accession Number |
| --- | --- | --- |
| Ribosomal protein S3 | S3 | AJ517687 |
| Elongation factor 1 | EF1 | AJ250733 |
| ß-Actin | BAct | AF082863 |
| Metallothionein | MT | U67347 |
| Heat-shock Protein 70 | HSP70 | EF526096 |
| Heat-shock Protein 90 | HSP90 | GU433881 |
| Glutathione S-transferase | GST | EF194203 |
| Superoxide dismutase | SOD | AY377970 |
| Glutathione peroxidase | GPx | DQ459994 |
| Catalase | CAT | EF681763 |
| Cytochrome c oxidase | COI | AM749000 |
| P-gycoprotein  (Multixenobiotic Resistance) | P-gp1 | AJ506742 |
